# *Polygonum multiflorum* Thunb. Extract Stimulates Melanogenesis by Induction of COX2 Expression through the Activation of p38 MAPK in B16F10 Mouse Melanoma Cells

**DOI:** 10.1155/2020/7642019

**Published:** 2020-06-25

**Authors:** Donghee Kim, Hyo-Jin Kim, Hee-Sook Jun

**Affiliations:** ^1^Lee Gil Ya Cancer and Diabetes Institute, Gachon University, Incheon 21999, Republic of Korea; ^2^College of Pharmacy, Gachon University, Incheon 21936, Republic of Korea; ^3^Gachon Medical and Convergence Institute, Gachon Gil Medical Center, Incheon 21565, Republic of Korea

## Abstract

*Polygonum multiflorum* Thunb. (PM) root extracts have been used for treating graying hair in Oriental medicine; however, the molecular mechanisms underlying the melanogenic effects of PM root have not been fully understood. In the present study, we investigated the melanogenic effects of an ethanolic extract of PM root (PME) and the mechanisms involved. We examined the effects of PME on cell viability, cellular melanin content, and tyrosinase activity in B16F10 cells. The melanogenic mechanism of PME was explored using signaling inhibitors and examining the expression of melanogenic genes and signaling molecules by western blot and RT-qPCR analyses. PME did not exhibit any cytotoxicity in B16F10 cells compared to that in control cells. PME treatment significantly increased melanin production and tyrosinase activity. In addition, PME induced the expression of cyclooxygenase-2 (COX2) as well as that of melanogenic genes, such as microphthalmia-associated transcription factor (MiTF), tyrosinase-related protein (Trp) 1, Trp2, and tyrosinase, in B16F10 cells. PME treatment increased the level of phosphorylated p38 mitogen-activated protein kinase (MAPK), and pretreatment with SB 203580, a p38 MAPK inhibitor, significantly suppressed this PME-induced increase in the expression of COX2 and melanogenic genes. These results indicate that PME induced the expression of melanogenic genes by inducing COX2 expression via the activation of the p38 MAPK pathway, thereby contributing to the enhancement of melanogenesis.

## 1. Introduction

Vitiligo is a skin disease characterized by the lack of pigmentation in the skin and white patches in the different parts of the body [1]. This disease affects 1% of the population of the world [2]. Most patients with vitiligo develop the disease at ages of 10–30 years. The pathophysiology of this disease has not been well understood; however, several causes have been identified, such as genetic, biochemical, immunological, and environmental factors [2]. Currently, the goal of vitiligo treatment is to suppress depigmentation and stimulate repigmentation [3]. Several treatments are used, including excimer lasers, vitamin D analogues, and steroid therapies that are aimed at restoring pigmentation; however, unfortunately the efficacy and safety of these treatments remain unsatisfactory and must be improved [4]. Therefore, novel agents for vitiligo diseases are needed.

Melanin is a natural pigment synthesized and stored in melanosomes of melanocytes [5]. The synthesis of melanin is regulated by three enzymes, tyrosinase, tyrosinase-related protein (Trp) 1, and Trp2, which is also known as dopachrome tautomerase (DCT) [6]. Tyrosinase is the key enzyme that regulates the rate-limiting step of melanin production, in which L-tyrosine is hydroxylated to L-3,4-dihydroxyphenylalanine (L-DOPA), and then L-DOPA can be converted into dopaquinone [7]. Furthermore, microphthalmia-associated transcription factor (MiTF) is a master regulator of the transcription of melanogenic genes [8, 9]. Major components of MiTF induction are the ultraviolet- (UV-) mediated induction of the proopiomelanocortin (POMC), *α*-melanocyte-stimulating hormone (*α*-MSH), stem cell factor (SCF), and endothelin-1 (ET-1) [10]. All of these factors induce melanogenesis through the following four main signaling pathways: (1) mitogen-activated protein kinase (MAPK) signaling pathway, (2) cyclic AMP (cAMP)/protein kinase A (PKA) signaling pathway, (3) phosphatidylinositol 3-kinase (PI3K)/protein kinase B (AKT) signaling pathway, and (4) Wnt signaling pathway [10].

Several medicinal plants have been reported to promote melanin production [11–14]. The root of the plant *Polygonum multiflorum* Thunb. (PM), known as “Ha Su O” in Korea (He Shou Wu in China), has also been used in Oriental medicine for the treatment of various diseases including hair aging [15, 16]. The extracts or active components of PM root have been reported to promote hair growth [16], strengthen hair follicle pigmentation [17, 18], and induce melanin production [11, 19]. Jiang and colleagues reported that 2,3,5,4′-tetrahydroxystilbene-2-O-beta-D-glucoside (THSG), a water-soluble active component extracted from PM root, induced melanogenesis by p38 MAPK activation and MiTF induction in B16 cells [19].

Cyclooxygenase-2 (COX2) is an enzyme that catalyzes the production of prostaglandin E2 (PGE2) in keratinocytes [20, 21]. PGE2 is important for the proliferation and melanogenesis of melanocytes [22, 23]. It has been reported that COX2 is also expressed in melanocytes [24], and the functional polymorphisms of COX2 have been associated with an increased risk of vitiligo [25]. More recently, it has been reported that siRNA-mediated downregulation of COX2 inhibits melanogenesis [26] and that COX2 is involved in inducing the expression of melanogenesis-related genes during melanogenesis [27]. Additionally, it is well known that various intracellular signaling proteins, including MAPK and PI3K/Akt, are involved in inducing COX2 expression [28]. However, the expression or relevance of COX2 with respect to the melanogenic effects of PM root extract have not been reported. Therefore, we investigated the involvement of COX2 in mediating the melanogenic effects of an ethanolic extract of PM root (PME) in B16F10 melanoma cells.

## 2. Materials and Methods

### 2.1. Chemicals

Isobutylmethylxanthine (IBMX), L-DOPA, melanin, SB 203580, p38 MAPK inhibitor, SP600125, JNK inhibitor, PD98059, Erk inhibitor, H-89, PKA Inhibitor, LY 294002, PI3K/Akt inhibitor, and all other chemicals were purchased from Sigma-Aldrich (St. Louis, MO, USA) unless otherwise indicated.

### 2.2. Preparation of PME

In this study, we used the 70% (v/v) ethanolic extracts of dried PM root (PME). The freeze-dried powder of PME (KOC201512-017) was purchased from an Oriental drug extraction store (KOC Biotech Co., Ltd., Daejeon, Korea). The stocks for the extracts were prepared by dissolving 10 mg of extract powder in 1 mL of dimethyl sulfoxide (DMSO; Duchefa Biochemie BV, Haarlem, Netherlands) and then stored at −20°C. Working concentrations were prepared by diluting the stock solutions with culture medium.

### 2.3. Cell Culture

B16F10 murine melanoma cells (from the Korean Cell Line Bank, Seoul, Korea) were cultured in Dulbecco's modified Eagle's medium (DMEM) (Welgene, Daegu, Korea) with 10% fetal bovine serum (FBS) (Gibco, Grand Island, NY, USA) and penicillin/streptomycin (100 IU/50 *μ*g/mL, Welgene) in a humidified atmosphere containing 5% CO_2_ at 37°C.

### 2.4. Cell Viability Assay

Cell viability was estimated by a D-Plus™ CCK assay kit (Dongin LS, Seoul, Korea) in accordance with the manufacturer's protocol. B16F10 cells (1 × 10^4^ cells/well) were seeded on 96-well plates. After 16–18 h of culture, the cells were treated with different final concentrations (1–40 *μ*g/mL) of PME for 24, 48, 72, and 96 h. Following incubation, the PME-treated medium was replaced with 100 *μ*L of CCK working solution, and the cells were further incubated at 37°C for 2 h. Thereafter, the optical density (OD) at 450 nm was determined using a VersaMax Microplate Reader (Molecular Devices, LLC, Sunnyvale, CA, USA). PME concentrations of 10 *μ*g/mL and lower were used in subsequent experiments.

### 2.5. Melanin Content Assay

The melanin content was measured as previously described [14], with slight modifications. B16F10 cells (5 × 10^4^ cells) were seeded overnight in a 60-mm dish. Subsequently, the cells were treated with different final concentrations (2.5–10 *μ*g/mL) of PME or IBMX. For inhibition of p38 MAPK signaling, the cells were treated with 10 *μ*M SB 203580 for 1 h prior to 10 *μ*g/mL PME treatment. The cells were cultured for 96 h; after that, the cells were harvested and the cell pellets prepared. The cell pellets were dissolved in 200 *μ*L of 1 N NaOH containing 10% DMSO for 1 h at 80°C. After centrifugation at 12,000*g* for 10 min at 25°C, the supernatant was collected. The cell lysate (100 *μ*L) was pipetted into a 96-well microplate (SPL Life Sciences, Gyeonggi-do, Korea), and the OD at 490 nm was determined using a VersaMax Microplate Reader. The OD values were normalized to the protein content in the cell lysates. Then the protein concentration was estimated by BCA Protein Assay kit (Pierce Biotechnology, Rockford, IL, USA) in accordance with the manufacturer's protocol.

### 2.6. Cellular Tyrosinase Activity

Tyrosinase activity was determined by measuring the L-DOPA oxidation rate [29] with some modifications. B16F10 cells (5 × 10^4^ cells) were seeded overnight in 60-mm dishes and then treated with different final concentrations (2.5–10 *μ*g/mL) of PME or IBMX. To inhibit p38 MAPK signaling, the cells were treated with 10 *μ*M SB 203580 for 1 h prior to 10 *μ*g/mL PME treatment. After 96 h incubation, the cells were washed with ice-cold Dulbecco's phosphate-buffered saline (DPBS) and lysed with PBS (50 mM, pH 6.8) containing 1% Triton X-100 and protease inhibitor cocktail (GenDEPOT Inc., Katy, TX, USA). The protein concentrations were then measured by BCA Protein Assay kit (Pierce Biotechnology), and the protein concentration in each cell lysate was equalized using lysis buffer. This was followed by the addition of 50 *μ*L of each lysate and 100 *μ*L of 5 mM L-DOPA to each well of a 96-well plate. The plate was kept at 37°C for 1 h in the dark and the absorbance was measured at 475 nm. Cells without PME or IBMX treatment served as the controls, and the relative activity of tyrosinase was expressed as (A_475_ of treatment/A_475_ of control) × 100%.

### 2.7. Western Blotting

B16F10 cells (3 × 10^5^ cells) were seeded on 60-mm dishes, incubated with vehicle (DMSO) or different final concentrations (2.5–10 *μ*g/mL) of PME for 24 h. For inhibition of p38 MAPK signaling, cells were treated with 10 *μ*M SB 203580 for 1 h prior to 10 *μ*g/mL PME treatment. To extract cellular proteins, the cells were lysed with Mammalian Protein Extraction Buffer (GE Healthcare, Milwaukee, WI, USA) containing a protease and phosphatase inhibitor cocktails (GenDEPOT Inc.). After centrifugation at 12,000*g* for 10 min at 4°C, the total proteins in the supernatants were quantified using the BCA assay. Five to thirty micrograms of protein were separated on a 10% SDS-PAGE gel and transferred onto polyvinylidene difluoride membranes (Millipore, Billerica, MA, USA). The membranes were then blocked with 5% skim milk in Tris-buffered saline containing 0.1% Tween 20 (TBST) for 1 h at 25°C, followed by incubation with the following primary antibodies overnight at 4°C: anti-tyrosinase, anti-MiTF, anti-phospho-p38 MAPK, anti-p38 MAPK (Cell Signaling Technology, Boston, MA, USA), anti-COX2, and anti-*β*-actin (Santa Cruz Biotechnology; Santa Cruz, CA, USA). After washing with TBST, the membranes were incubated with horseradish peroxidase-conjugated secondary antibodies (Santa Cruz Biotechnology) for 1 h at 25°C. Chemiluminescent signals were developed by treating the blot with ECL reagents, WESTSAVE (AbFrontier, Seoul, Korea), and visualized using the LAS4000 imaging system (Fujifilm Corp.; Tokyo, Japan). The intensities of the target protein bands were quantified using ImageJ (National Institutes of Health, Bethesda, MD, USA).

### 2.8. RNA Isolation and Quantitative Real-Time PCR (RT-qPCR)

B16F10 cells (3 × 10^5^ cells) were seeded overnight in a 60-mm dish. Then, cells were treated with a final concentration (10 *μ*g/mL) of PME for 6 h (for evaluation of MiTF, Trp1, and Trp2) or 24 h (for evaluation of tyrosinase). Total RNA isolation and RT-qPCR were performed by following the methods in our previous report [30]. In brief, total RNA was extracted using RNAiso Plus Reagent (TaKaRa Bio Inc., Shiga, Japan), and 2 *μ*g of total RNA was reverse-transcribed using a PrimeScript™ 1st strand cDNA synthesis kit (TaKaRa Bio Inc.). RT-qPCR was performed using a reaction mixture comprising SYBR Green master mix (TaKaRa Bio Inc.). Results were calculated using the 2^−ΔΔCT^ relative quantification method and normalized to the expression level of cyclophilin B. The sequences of the primer pairs are shown in Table 1.

### 2.9. Immunocytochemistry

B16F10 cells (2 × 10^4^ cells/well) were seeded overnight in the Nunc™ Lab-Tek™ II Chamber Slide™ system (Thermo Fisher Scientific, Rochester, NY, USA). The cells were pretreated with vehicle (DMSO) or 10 *μ*M SB 203580 for 1 h and then further treated with or without a final concentration of 10 *μ*g/mL of PME for 24 h. The cells were washed twice with DPBS and then fixed in 4% paraformaldehyde for 15 min at 25°C. After another wash with DPBS, the cells were blocked in Protein Block Serum-Free Ready-to-Use solution (Dako North America, Inc., Carpinteria, CA, USA) at 25°C for 1 h, followed by incubation overnight with anti-tyrosinase (Cell Signaling Technology) antibody diluted in Antibody Diluent (1 : 200, Dako North America, Inc.) at 4°C. The cells were then stained with Alexa Fluor 546-conjugated secondary antibody (1 : 200, Life Technologies, Carlsbad, CA, USA) at 25°C for 2 h. The nuclei were stained with 4′,6-diamidino-2-phenylindole (DAPI; Invitrogen, San Diego, CA, USA) diluted in DPBS (1 : 1000) at 25°C for 5 min. Cells were mounted with Fluorescence Mounting Medium (Dako North America, Inc.) and then observed under a confocal microscope (LSM700; Carl Zeiss Inc., Oberkochen, Germany).

### 2.10. Statistical Analysis

All data are presented as mean ± standard error of the mean (SEM). Statistical analyses were performed using GraphPad Prism version 5 (GraphPad Software Inc., San Diego, CA, USA). Data were analyzed using Student's *t*-test for comparisons between two groups, and one- or two-way analysis of variance (ANOVA) followed by Tukey's multiple comparison or Bonferroni tests, as appropriate, for comparisons among more than two groups. *p* < 0.05 was considered statistically significant.

## 3. Results

### 3.1. PME Enhances Melanin Production and Tyrosinase Activity in B16F10 Cells

**Figure 1:**
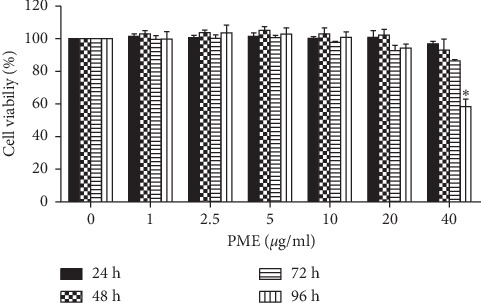
Effect of PME on B16F10 cell viability. Cells were treated with different final concentrations (1, 2.5, 5, 10, 20, and 40 *μ*g/mL) of PME for 24, 48, 72, and 96 h. Cell viability was determined using CCK8 assay kit and calculated relative to that of control cells. Control: vehicle only. Data are presented as mean ± SEM of three independent experiments.

To determine whether PME has any cytotoxic effects on B16F10 cells, we first examined the cell viability of B16F10 cells after treatment with different final concentrations of PME (1–40 *μ*g/mL) for 24, 48, 72, and 96 h by the CCK8 assay. As shown in Figure 1, there was no difference in viability between the control and PME-treated cells, except for the treatment with 40 *μ*g/mL of PME for 96 h (Figure 1). These results indicate that the concentrations of PME used in this study do not have any cellular toxicity against B16F10 cells.

**Figure 2:**
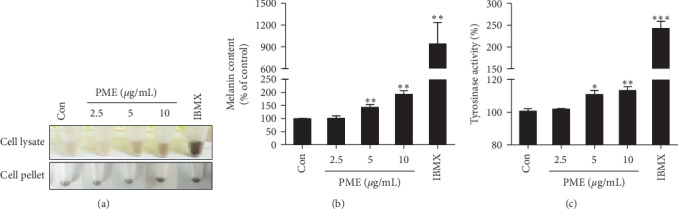
PME treatment enhanced melanin production and tyrosinase activity in B16F10 cells. B16F10 cells were treated with 2.5, 5, and 10 *μ*g/mL PME or with 100 *μ*M IBMX for 96 h. (a) Representative images of cell lysates and pellets of B16F10 cells after incubation. (b) Absorbance of 490 nm was measured in each cell lysate, and melanin levels were calculated relative to those of control cells. (c) Absorbance of 475 nm, indicating the L-DOPA oxidation rate, was measured in each cell lysate, and tyrosinase activity was calculated relative to that of control cells. Control (Con): vehicle only. Data are presented as mean ± SEM. ^*∗*^*p* < 0.05, ^*∗∗*^*p* < 0.01, ^*∗∗∗*^*p* < 0.001 vs. control.

To determine the effects of PME on melanin production, we treated B16F10 cells with different final concentrations of PME (2.5, 5, and 10 *μ*g/mL) for 24, 48, 72, and 96 h and found that melanin content was the highest in cells treated with PME for 96 h (data not shown). Therefore, we treated B16F10 cells with PME for 96 h for the melanin content assay. IBMX was used as a positive control. As shown in Figure 2(a), the color of cell lysates and pellets darkened in a dose-dependent manner in response to PME treatment (Figure 2(a)). Consistent with this finding, treatment with PME resulted in a dose-dependent increase in melanin levels (Figure 2(b)). Melanin production is known to be regulated by tyrosinase [31]. Therefore, we next investigated whether tyrosinase was involved in PME-induced melanin production. Similarly to melanin content, tyrosinase activity was also the highest when we treated cells with PME for 96 h in our preliminary experiments (data not shown). As shown in Figure 2(c), the tyrosinase activity was significantly increased following PME treatment in a dose-dependent manner (Figure 2(c)). All these results suggest that PME enhances melanin production by increasing tyrosinase activity in B16F10 cells.

### 3.2. PME Increases the Expression of Melanogenesis-Related Genes in B16F10 Cells

**Figure 3:**
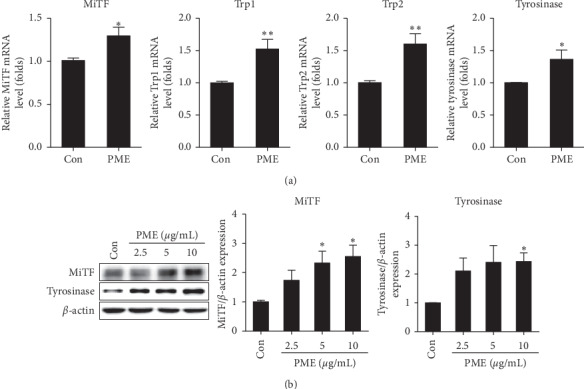
PME treatment increased expression of melanogenesis-related genes in B16F10 cells. (a) B16F10 cells were treated with the PME (10 *μ*g/mL) for 6 h (for evaluation of MiTF, Trp1, and Trp2) or 24 h (for evaluation of tyrosinase). The mRNA levels of melanogenesis-related genes were measured by RT-qPCR. (b) B16F10 cells were treated with PME (2.5, 5, and 10 *μ*g/mL) for 24 h. The protein levels of MiTF and tyrosinase were measured by western blotting. The relative expression levels of the proteins were normalized to that of *β*-actin and quantified using ImageJ. Control: vehicle only. Data are presented as mean ± SEM of three to four independent experiments. ^*∗*^*p* < 0.05, ^*∗∗*^*p* < 0.01 vs. control.

We then examined mRNA expression of MiTF, Trp1, Trp2, and tyrosinase, which are known to be involved in melanogenesis [6–9], in PME-treated B16F10 cells by RT-qPCR. As shown in Figure 3(a), the expression levels of MiTF, Trp1, and Trp2 mRNA were significantly increased following treatment with 10 *μ*g/mL PME for 6 h, and the levels of tyrosinase mRNA were also increased following treatment with 10 *μ*g/mL PME for 24 h in B16F10 cells (Figure 3(a)). Additionally, MiTF and tyrosinase protein levels were also significantly increased by PME treatment in a dose-dependent manner (Figure 3(b)). These results indicate that PME induces melanogenesis through the upregulation of the expression of MiTF and tyrosinase.

### 3.3. PME Increases COX2 Expression in Association with the Increase of Phosphorylated p38 MAPK

**Figure 4:**
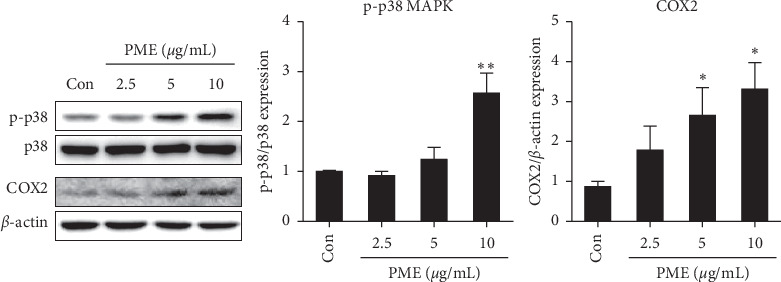
PME treatment increased the phosphorylation of p38 MAPK and the expression of COX2 in B16F10 cells. B16F10 cells were treated with PME (2.5, 5, and 10 *μ*g/mL) for 24 h. The protein levels in each cell lysate were estimated by western blotting. The relative expression levels of the p-p38 and COX2 were normalized to those of p38 and *β*-actin, respectively, and were quantified using ImageJ. Control (Con): vehicle only. Data are presented as mean ± SEM of three to four independent experiments. ^*∗*^*p* < 0.05, ^*∗∗*^*p* < 0.01 vs. control.

As mentioned above, melanogenesis is regulated via four main signaling pathways, all of which involve induction of MiTF [10]. To investigate the signaling pathways underlying the MiTF induction by PME, we first examined the changes of melanin production by PME using chemical inhibitors of major signaling pathways such as LY 294002 (PI3K/Akt inhibitor), H-89 (PKA inhibitor), SP600125 (JNK inhibitor), PD 98059 (Erk inhibitor), and SB 203580 (p38 MAPK inhibitor). Pretreatment with SB 203580 inhibited PME-induced melanin production. However, pretreatment with Ly 294002, H-89, SP600125, or PD 98059 did not change PME-induced melanin production in B16F10 cells (data not shown). Therefore, we checked whether PME treatment actually activates p38 MAPK and found that the expression of phospho-p38 MAPK was significantly increased upon PME treatment in a dose-dependent manner (Figure 4).

It was reported that COX2 is involved in inducing the expression of melanogenesis-related genes [26, 27], and COX2 expression is regulated by p38 MAPK activation in HaCaT cells [32]. Therefore, we then investigated whether the expression of COX2 was increased by PME treatment in B16F10 cells. COX2 expression was also significantly increased by PME treatment in a dose-dependent manner (Figure 4).

### 3.4. PME Induces COX2 Expression via Activation of p38 MAPK, Contributing to the Melanogenesis in B16F10 Cells

**Figure 5:**
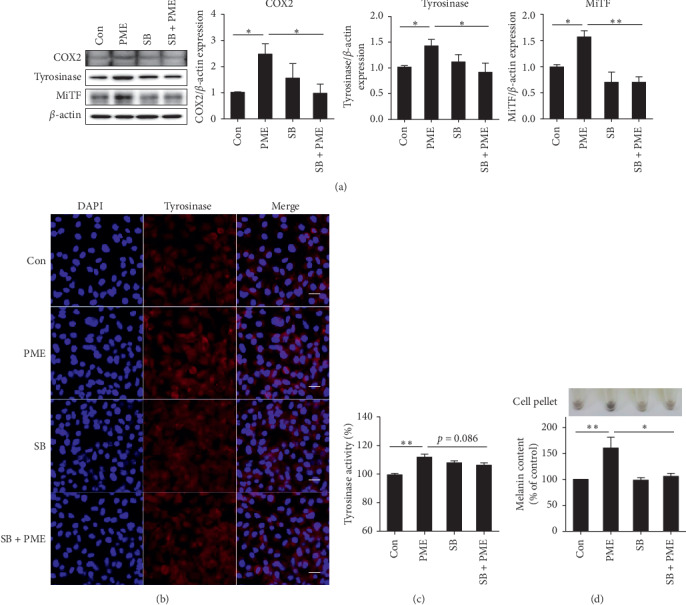
PME treatment enhanced melanogenesis by induction of COX2 expression through the activation of p38 MAPK in B16F10 cells. (a) B16F10 cells were pretreated with 10 *μ*M SB 203580 (SB) for 1 h and then treated with 10 *μ*g/mL of PME for 24 h. The protein levels of COX2, tyrosinase, and MiTF were estimated by western blotting. The relative expression levels of the target proteins were normalized to that of *β*-actin and quantified using the ImageJ software. Control (Con): vehicle only. Data are presented as mean ± SEM of three to four independent experiments. ^*∗*^*p* < 0.05, ^*∗∗*^*p* < 0.01 vs. control or PME. (b) B16F10 cells were seeded in 4-well chambers and pretreated with 10 *μ*M SB for 1 h, followed by further treatment with 10 *μ*g/mL PME for 24 h. Immunocytochemistry was performed using an anti-tyrosinase antibody and Alexa Fluor 546-conjugated secondary antibody. The nuclei were counterstained with DAPI (blue) (original magnification, 400x; scale bars represent 20 *μ*m). (c, d) B16F10 cells were pretreated with 10 *μ*M SB for 1 h and then treated with 10 *μ*g/mL PME for 96 h. The tyrosinase activity (c) and melanin content (d) in cell lysates were analyzed by measuring the absorbance at 475 nm and 490 nm, respectively. Data are presented as the mean ± SEM of three to four independent experiments. ^*∗*^*p* < 0.05, ^*∗∗*^*p* < 0.01 vs. control or PME.

To investigate whether the PME-induced activation of p38 MAPK increases COX2 expression, we examined the expression of COX2 in B16F10 cells under pretreatment with SB 203580. The expression of COX2 was significantly increased in cells treated with PME compared to that in control cells. However, the expression of COX2 protein was significantly decreased in SB 203580-pretreated cells relative to that in PME-treated cells (Figure 5(a)). In parallel, the expression of tyrosinase and MiTF was also increased by PME treatment, and this increase was inhibited by pretreatment with SB 203580 (Figure 5(a)). Immunofluorescence staining with anti-tyrosinase antibody showed that treatment with PME increased the fluorescence intensity in the cytoplasm of B16F10 cells compared with that in the control (Con) group, and this increased fluorescence intensity was reduced by pretreatment with SB 203580 (SB + PME) (Figure 5(b)). This indicates that the expression level of tyrosinase was increased by treatment with PME; however, treatment with SB 203580 suppressed this effect. Consistent with these results, PME-induced tyrosinase activity tended to decrease in SB 203580-pretreated B16F10 cells (Figure 5(c)). Finally, we found that SB 203580 pretreatment significantly inhibited PME-induced pellet darkening and melanin contents in B16F10 cells (Figure 5(d)). Similarly, PME increased melanin production and melanogenic gene expression in a concentration-dependent manner in human melanoma cell line, SK-MEL-28 cells, and the increased melanin production and expression of melanogenic genes by PME were suppressed by pretreatment of SB 203580 (Supplementary Figure 1). Collectively, these results indicate that PME induces melanogenesis through induction of COX2 expression via activation of the p38 MAPK signaling pathway.

## 4. Discussion

PM root has been used in Oriental medicine and exhibits low toxicity as well as various pharmacological activities, such as liver and kidney detoxification and antiallergic, antitumor, antibacterial, and antiaging effects [15, 16]. More recently, the extract of PM root has been reported to have protective effects on diabetes-induced bone loss [33], hepatoprotective activity [34], and antiobesity effects [35]. Importantly, the extracts or active components, such as THSG, of PM root have also been reported to promote hair growth [16], enhance hair follicle pigmentation [17, 18], and induce melanin production [11, 19], which imply a potential as a treatment for vitiligo. However, the molecular mechanisms have not been fully understood. In this study, we focused on determining the mechanism by which PME increases melanogenesis, and demonstrated that PME induces melanogenesis by increasing COX2 expression through the p38 MAPK pathway in B16F10 cells.

The extract of PM root has been reported to exert protective effects in hepatocytes (20–100 *μ*g/mL for 48 h) [34] and hippocampal cells (0.1–10 *μ*g/mL for 24 h) [36]; however, extracts can be toxic depending on the extraction method and cell type. Thus, we first confirmed the effects on cell viability in B16F10 cells (Figure 1). PME doses ranging from 1 to 20 *μ*g/mL for 96 h did not affect the viability of B16F10 cells.

PME promoted melanin production, tyrosinase activity, and the expression of melanogenic genes, such as MiTF and tyrosinase (Figures 2 and 3) in B16F10 cells, as previously reported in mouse B16 cells [11, 19], rat dermal papilla cells [16], human foreskin melanocytes [17], and human melanoma SK-MEL-18 cells and zebrafish [18]. When we tested several signaling inhibitors, such as LY 294002 for the PI3K/Akt pathway, H-89 for the PKA pathway, SP 600125 for the JNK pathway, PD 98059 for the Erk pathway, and SB 203580 for the p38 MAPK pathway, only SB 203580 inhibited PME-induced melanogenesis. Similar to the results observed in B16F10 cells, we also confirmed the melanogenic effect of PME on human melanoma cell line, SK-MEL-28 cells, and this effect was mediated by p38 MAPK activation (Supplementary Figure 1). Consistent with our results, it was reported that THSG, an active component of PM root extract, resulted in increased melanogenesis in B16 cells through p38 MAPK activation, but not Erk or JNK activation [19]. However, THSG has been reported to exert its neuroprotective effects through the inhibition of p38 MAPK activation [37] and activation of PI3K/Akt signaling pathway [38] in PC12 cells. In addition, emodin, another active component of PM root, inhibited melanogenesis by promoting the degradation of MiTF by Erk activation in human melanocytes [39]. Therefore, the functional effects and signaling mechanisms are dependent on the active ingredient of the PM root, and even the same active ingredient can act through different signaling mechanisms depending on the cell type.

Exposure of intact skin to UV radiation results in melanogenesis [40]; therefore, narrowband UV-B (311–312 nm) is widely used as the first choice of phototherapy for patients with vitiligo [41]. UVB light increases the expression of COX2 and PGE2 in the skin [42]. COX2 is an enzyme that catalyzes the production of PGE2 in keratinocytes [20, 21]. Additionally, Li M and colleagues reported that polymorphisms in the COX2 gene are associated with an increased risk of vitiligo [25]. In general, studies on the roles of COX2 and PGE2 in the skin have focused on keratinocytes. PGE2 secreted from keratinocytes acts as a paracrine factor and plays an important role in the regulation of melanocyte function [26, 43]. Furthermore, PGE2 produced by COX2 in keratinocytes induced MiTF expression and finally melanin production in melanocyte [43]. However, it has been reported that melanocytes also produce PGE2 in response to UVB [24]. UVB-induced PGE2, an autocrine factor, stimulates tyrosinase in melanocyte [23]. The siRNA-mediated knock-down of COX2 in melanocytes suppressed the expression of melanogenic factors, such as MiTF, Trp1, Trp2, and tyrosinase, even in melanogenic stimulation conditions [26]. More recently, nonselective COX inhibitors, including indomethacin, have been reported to inhibit melanogenesis [44, 45]. Thus, induction of COX2 expression appears to play an important role in promoting melanin production. Therefore, in this study, we evaluated the effects of PME on COX2 expression in B16F10 melanocytes. We found for the first time that PME significantly increased COX2 expression without increasing cell proliferation in B16F10 melanocytes (Figures 1 and 4). These results suggest that PME directly acts to induce COX2 expression and subsequently PGE2 production, which may play an important role in enhancing melanogenesis in melanocytes.

UVB light induces COX2 expression in skin [20] by increased phosphorylation of CREB at serine 133 through the activation of p38 MAPK [46, 47]. Similarly, treatment with p38 MAPK inhibitor reversed the upregulation of COX2 by PME, indicating that PME treatment induces COX2 expression via p38 MAPK activation in melanocytes.

In summary, melanocytes treated with PME exhibited increased expression of MiTF, Trp1, Trp2, and tyrosinase, as well as enhanced melanin production and tyrosinase activity. PME treatment resulted in increased phosphorylation of p38 MAPK and expression of COX2. Furthermore, SB 203580, a p38 MAPK inhibitor, reduced PME-induced melanin production and COX2 expression. These results suggest that PME induced the expression of melanogenic genes by inducing COX2 expression via the activation of the p38 MAPK pathway, thereby contributing to the enhancement of melanogenesis.

## Figures and Tables

**Table 1:** Primer sets used for RT-qPCR analyses.

Gene name	Sense primer (5′-3′)	Antisense primer (5′-3′)
Cyclophilin B	TGG AGA GCA CCA AGA CAG ACA	TGC CGG AGT CGA CAA TGA T
MiTF	AAC AAG GGA ACC ATT CTC AAG G	TGC TCC AGC TTC TTC TGT CG
Trp1	GCT GCA GGA GCC TTC TTT CTC	GTC ATC AGT GCA GAC ATC GC
Trp2	AAC AGA CAC CAG ACC CTG GA	AAG TTT CCT GTG CAT TTG CAT GT
Tyrosinase	CTC TGG GCT TAG CAG TAG GC	GCA AGC TGT GGT AGT CGT CT

## Data Availability

The data used to support the findings of this study are available from the corresponding author upon request.

## Abbreviations

AKT: Protein kinase B
*α*-MSH: *α*-Melanocytes stimulating hormone
cAMP: Cyclic AMP
COX2: Cyclooxygenase-2
DCT: Dopachrome tautomerase
DMEM: Dulbecco's modified Eagle's medium
DMSO: Dimethyl sulfoxide
ET-1: Endothelin-1
FBS: Fetal bovine serum
IBMX: Isobutylmethylxanthine
L-DOPA: L-3,4-dihydroxyphenylalanine
MAPK: Mitogen-activated protein kinase
MC1R: Melanocortin-1 receptor
MiTF: Microphthalmia-associated transcription factor
OD: Optical density
PGE2: Prostaglandin E2
PI3K: Phosphatidylinositol 3-kinase
PKA: Protein kinase A
PME: 70% ethanolic extract of *Polygonum multiflorum* Thunb. root
SCF: Stem cell factor
THSG: 2,3,5,4′-Tetrahydroxystilbene-2-O-beta-D-glucoside
Trp1: Tyrosinase-related protein 1
Trp2: Tyrosinase-related protein 2.

## Supplementary Materials

Supplementary Materials Supplementary Figure 1: PME induces melanogenesis through activation of p38 MAPK in SK-MEL-28 cells. (a) Effect of PME on SK-MEL-28 cell viability. Cells (1 × 10 4 cells/well) were treated with different final concentrations (37.5, 75, 150, 300, and 600 *μ*g/mL) of PME for 48 h. Cell viability was determined using CCK8 assay kit and calculated relative to that of control cells. Control (Con): vehicle only. Data are presented as mean ± SEM of triplicate experiments. (b) SK-MEL-28 cells (5 × 10 5 cells/dish) were treated with 75, 150, and 300 *μ*g/mL PME for 48 h. Representative images of cell pellets of SK-MEL-28 cells after incubation. Absorbance of 490 nm was measured in each cell lysate, and melanin levels were calculated relative to those of control cells. (c) SK-MEL-28 cells (5 × 10 5 cells/dish) were treated with 75, 150, and 300 *μ*g/mL PME for 24 h. The protein levels of tyrosinase, MiTF, p-p38, p38, COX2, and *β*-actin were measured by western blotting. Representative blots are shown. (d, e) SK-MEL-28 cells (5 × 10 5 cells/dish) were pretreated with 10 *μ*M SB 203580 (SB) for 1 h and then treated with 300 *μ*g/mL of PME for 24 h. (d) Representative images of cell pellets of SK-MEL-28 cells after incubation. The melanin content (d) in cell lysates was analyzed by measuring the absorbance at 490 nm, and melanin levels were calculated relative to those of control cells. (e) The protein levels of COX2, tyrosinase, and MiTF were measured by western blotting. Representative blots are shown. Data are presented as mean ± SEM. ^*∗*^*p* < 0.05, ^*∗∗*^*p* < 0.01 vs. control or PME.

## References

[1] Ezzedine K., Eleftheriadou V., Whitton M., van Geel N. (2015). Vitiligo. *The Lancet*.

[2] Whitton M., Pinart M., Batchelor J. M. (2016). Evidence-based management of vitiligo: summary of a cochrane systematic review. *British Journal of Dermatology*.

[3] Birlea S. A., Goldstein N. B., Norris D. A. (2017). Repigmentation through melanocyte regeneration in vitiligo. *Dermatologic Clinics*.

[4] Picardo M., Dell’Anna M. L., Ezzedine K. (2015). Vitiligo. *Nature Reviews Disease Primers*.

[5] Tobin D. J. (2011). The cell biology of human hair follicle pigmentation. *Pigment Cell & Melanoma Research*.

[6] Tuerxuntayi A., Liu Y. Q., Tulake A., Kabas M., Eblimit A., Aisa H. A. (2014). Kaliziri extract upregulates tyrosinase, TRP-1, TRP-2 and MITF expression in murine B16 melanoma cells. *BMC Complementary and Alternative Medicine*.

[7] D’Mello S. A., Finlay G. J., Baguley B. C., Askarian-Amiri M. E. (2016). Signaling pathways in melanogenesis. *International Journal of Molecular Sciences*.

[8] Kawakami A., Fisher D. E. (2017). The master role of microphthalmia-associated transcription factor in melanocyte and melanoma biology. *Laboratory Investigation*.

[9] Levy C., Khaled M., Fisher D. E. (2006). MITF: master regulator of melanocyte development and melanoma oncogene. *Trends in Molecular Medicine*.

[10] Niu C., Aisa H. A. (2017). Upregulation of melanogenesis and tyrosinase activity: potential agents for vitiligo. *Molecules*.

[11] Guan S., Su W., Wang N., Li P., Wang Y. (2008). A potent tyrosinase activator from radix polygoni multiflori and its melanogenesis stimulatory effect in B16 melanoma cells. *Phytotherapy Research*.

[12] Moreira C. G., Horinouchi C. D., Souza-Filho C. S. (2012). Hyperpigmentant activity of leaves and flowers extracts of *Pyrostegia venusta* on murine B16F10 melanoma. *Journal of Ethnopharmacology*.

[13] Wang J. Y., Chen H., Wang Y. Y. (2017). Network pharmacological mechanisms of *Vernonia anthelmintica* (L.) in the treatment of vitiligo: isorhamnetin induction of melanogenesis via up-regulation of melanin-biosynthetic genes. *BMC Systems Biology*.

[14] Yao C., Jin C. L., Oh I. G., Park C. H., Chung J. H. (2015). Melia azedarach extract stimulates melanogenesis through increase of tyrosinase-related protein 1 expression in B16F10 mouse melanoma cells. *International Journal of Molecular Medicine*.

[15] Lin L., Ni B., Lin H. (2015). Traditional usages, botany, phytochemistry, pharmacology and toxicology of *Polygonum multiflorum* Thunb.: a review. *Journal of Ethnopharmacology*.

[16] Sun Y. N., Cui L., Li W. (2013). Promotion effect of constituents from the root of *Polygonum multiflorum* on hair growth. *Bioorganic & Medicinal Chemistry Letters*.

[17] Sextius P., Betts R., Benkhalifa I. (2017). *Polygonum multiflorum* radix extract protects human foreskin melanocytes from oxidative stress in vitro and potentiates hair follicle pigmentation ex vivo. *International Journal of Cosmetic Science*.

[18] Thang N. D., Diep P. N., Lien P. T., Lien L. T. (2017). *Polygonum multiflorum* root extract as a potential candidate for treatm ent of early graying hair. *Journal of Advanced Pharmaceutical Technology & Research*.

[19] Jiang Z., Xu J., Long M., Tu Z., Yang G., He G. (2009). 2, 3, 5, 4’-tetrahydroxystilbene-2-O-beta-D-glucoside (THSG) induces melanogenesis in B16 cells by MAP kinase activation and tyrosinase upregulation. *Life Sciences*.

[20] Buckman S. Y., Gresham A., Hale P. (1998). COX-2 expression is induced by UVB exposure in human skin: implications for the development of skin cancer. *Carcinogenesis*.

[21] Smith W. L., Marnett L. J. (1991). Prostaglandin endoperoxide synthase: structure and catalysis. *Biochimica et Biophysica Acta*.

[22] Nordlund J. J., Collins C. E., Rheins L. A. (1986). Prostaglandin E2 and D2 but not MSH stimulate the proliferation of pigment cells in the pinnal epidermis of the DBA/2 mouse. *Journal of Investigative Dermatology*.

[23] Starner R. J., McClelland L., Abdel-Malek Z., Fricke A., Scott G. (2010). PGE(2) is a UVR-inducible autocrine factor for human melanocytes that stimulates tyrosinase activation. *Experimental Dermatology*.

[24] Gledhill K., Rhodes L. E., Brownrigg M. (2010). Prostaglandin-E2 is produced by adult human epidermal melanocytes in response to UVB in a melanogenesis-independent manner. *Pigment Cell & Melanoma Research*.

[25] Li M., Gao Y., Li C. (2009). Association of COX2 functional polymorphisms and the risk of vitiligo in Chinese populations. *Journal of Dermatological Science*.

[26] Kim J. Y., Shin J. Y., Kim M. R., Hann S. K., Oh S. H. (2012). siRNA-mediated knock-down of COX-2 in melanocytes suppresses melanogenesis. *Experimental Dermatology*.

[27] Eo S. H., Kim S. J. (2019). Resveratrol-mediated inhibition of cyclooxygenase-2 in melanocytes suppresses melanogenesis through extracellular signal-regulated kinase 1/2 and phosphoinositide 3-kinase/Akt signalling. *European Journal of Pharmacology*.

[28] Hong H., Park Y. K., Choi M. S. (2009). Differential down-regulation of COX-2 and MMP-13 in human skin fibroblasts by glucosamine-hydrochloride. *Journal of Dermatological Science*.

[29] Sarkar C., Singh S. K., Mandal S. K. (2006). Human placental protein/peptides stimulate melanin synthesis by enhancing tyrosinase gene expression. *Molecular and Cellular Biochemistry*.

[30] Kim D., Li H. Y., Lee J. H., Oh Y. S., Jun H. S. (2019). Lysophosphatidic acid increases mesangial cell proliferation in models of diabetic nephropathy via Rac1/MAPK/KLF5 signaling. *Experimental & Molecular Medicine*.

[31] Casanola-Martin G. M., Le-Thi-Thu H., Marrero-Ponce Y. (2014). Tyrosinase enzyme: 1. An overview on a pharmacological target. *Current Topics in Medicinal Chemistry*.

[32] Cho J. W., Park K., Kweon G. R. (2005). Curcumin inhibits the expression of COX-2 in UVB-irradiated human keratinocytes (HaCaT) by inhibiting activation of AP-1: p38 MAP kinase and JNK as potential upstream targets. *Experimental & Molecular Medicine*.

[33] Ham J. R., Lee H. I., Choi R. Y. (2019). Heshouwu (*Polygonum multiflorum* Thunb.) extract attenuates bone loss in diabetic mice. *Preventive Nutrition and Food Science*.

[34] Lin E. Y., Chagnaadorj A., Huang S. J., Wang C. C., Chiang Y. H., Cheng C. W. (2018). Hepatoprotective activity of the ethanolic extract of *Polygonum multiflorum* Thunb. against oxidative stress-induced liver injury. *Evidence-Based Complementary and Alternative Medicine*.

[35] Choi R. Y., Lee H. I., Ham J. R., Yee S. T., Kang K. Y., Lee M. K. (2018). Heshouwu (*Polygonum multiflorum* Thunb.) ethanol extract suppresses pre-adipocytes differentiation in 3T3-L1 cells and adiposity in obese mice. *Biomedicine & Pharmacotherapy*.

[36] Kim H. N., Kim Y. R., Jang J. Y. (2013). Neuroprotective effects of *Polygonum multiflorum* extract against glutamate-induced oxidative toxicity in HT22 hippocampal cells. *Journal of Ethnopharmacology*.

[37] Zhang L., Huang L., Li X. (2017). Potential molecular mechanisms mediating the protective effects of tetrahydroxystilbene glucoside on MPP(+)-induced PC12 cell apoptosis. *Molecular and Cellular Biochemistry*.

[38] Qin R., Li X., Li G. (2011). Protection by tetrahydroxystilbene glucoside against neurotoxicity induced by MPP+: the involvement of PI3K/Akt pathway activation. *Toxicology Letters*.

[39] Kim M. O., Park Y. S., Nho Y. H. (2016). Emodin isolated from polygoni multiflori ramulus inhibits melanogenesis through the liver X receptor-mediated pathway. *Chemico-Biological Interactions*.

[40] Tran T. T., Schulman J., Fisher D. E. (2008). UV and pigmentation: molecular mechanisms and social controversies. *Pigment Cell & Melanoma Research*.

[41] Scherschun L., Kim J. J., Lim H. W. (2001). Narrow-band ultraviolet B is a useful and well-tolerated treatment for vitiligo. *Journal of the American Academy of Dermatology*.

[42] Lotti T. M., Hercogova J., Schwartz R. A. (2012). Treatments of vitiligo: what’s new at the horizon. *Dermatologic Therapy*.

[43] Lee H. S., Goh M. J., Kim J. (2015). A systems-biological study on the identification of safe and effective molecular targets for the reduction of ultraviolet B-induced skin pigmentation. *Scientific Reports*.

[44] Sato K., Takahashi H., Toriyama M. (2011). Depigmenting mechanism of NSAIDs on B16F1 melanoma cells. *Archives of Dermatological Research*.

[45] Sato K., Takei M., Iyota R., Muraoka Y., Nagashima M., Yoshimura Y. (2017). Indomethacin inhibits melanogenesis via down-regulation of Mitf mRNA transcription. *Bioscience, Biotechnology, and Biochemistry*.

[46] Bowden G. T. (2004). Prevention of non-melanoma skin cancer by targeting ultraviolet-B-light signalling. *Nature Reviews Cancer*.

[47] Chen W., Tang Q., Gonzales M. S., Bowden G. T. (2001). Role of p38 MAP kinases and ERK in mediating ultraviolet-B induced cyclooxygenase-2 gene expression in human keratinocytes. *Oncogene*.

