# Supplementary material for: Polygonum multiflorum Thunb. Extract Stimulates Melanogenesis by Induction of COX2 Expression through the Activation of p38 MAPK in B16F10 Mouse Melanoma Cells
Source: Evid Based Complement Alternat Med. 2020 Jun 25;2020:7642019. doi: 10.1155/2020/7642019 (PMC7334760; doi:10.1155/2020/7642019)
Supplement: Supplementary Materials — Supplementary Figure 1: PME induces melanogenesis through activation of p38 MAPK in SK-MEL-28 cells. (a) Effect of PME on SK-MEL-28 cell viability. Cells (1 × 10 4 cells/well) were treated with different final concentrations (37.5, 75, 150, 300, and 600 μg/mL) of PME for 48 h. Cell viability was determined using CCK8 assay kit and calculated relative to that of control cells. Control (Con): vehicle only. Data are presented as mean ± SEM of triplicate experiments. (b) SK-MEL-28 cells (5 × 10 5 cells/dish) were treated with 75, 150, and 300 μg/mL PME for 48 h. Representative images of cell pellets of SK-MEL-28 cells after incubation. Absorbance of 490 nm was measured in each cell lysate, and melanin levels were calculated relative to those of control cells. (c) SK-MEL-28 cells (5 × 10 5 cells/dish) were treated with 75, 150, and 300 μg/mL PME for 24 h. The protein levels of tyrosinase, MiTF, p-p38, p38, COX2, and β-actin were measured by western blotting. Representative blots are shown. (d, e) SK-MEL-28 cells (5 × 10 5 cells/dish) were pretreated with 10 μM SB 203580 (SB) for 1 h and then treated with 300 μg/mL of PME for 24 h. (d) Representative images of cell pellets of SK-MEL-28 cells after incubation. The melanin content (d) in cell lysates was analyzed by measuring the absorbance at 490 nm, and melanin levels were calculated relative to those of control cells. (e) The protein levels of COX2, tyrosinase, and MiTF were measured by western blotting. Representative blots are shown. Data are presented as mean ± SEM. ∗p < 0.05, ∗∗p < 0.01 vs. control or PME. [file 7642019.f1.docx]

**Evidence-Based Complementary and Alternative Medicine**

**Manuscript #: 7642019**

***Polygonum multiflorum* Thunb. extract stimulates melanogenesis by induction of COX2 expression through the activation of p38 MAPK in B16F10 mouse melanoma cells**

**Supplementary Figure**


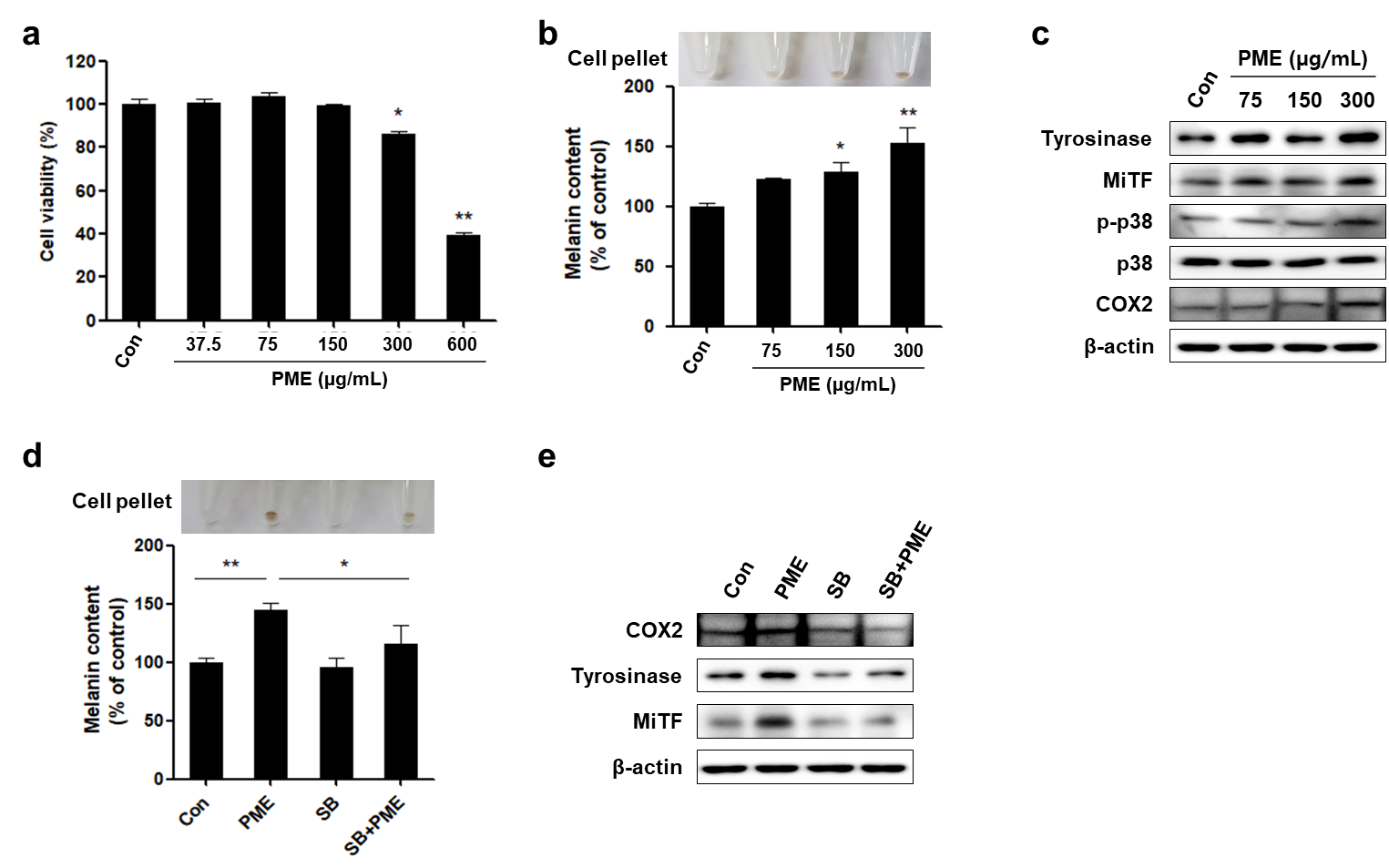


**Supplementary Figure 1. PME induces melanogenesis through activation of p38 MAPK in SK-MEL-28 cells.** (a) Effect of PME on SK-MEL-28 cell viability. Cells (1ⅹ10^4^ cells/well) were treated with different final concentrations (37.5, 75, 150, 300, and 600 μg/mL) of PME for 48 h. Cell viability was determined using CCK8 assay kit and calculated relative to that of control cells. Control (Con), vehicle only. Data are presented as the mean ± SEM of triplicate experiments. (b) SK-MEL-28 cells (5ⅹ10^5^ cells/dish) were treated with 75, 150, and 300 µg/mL PME for 48 h. Representative images of cell pellets of SK-MEL-28 cells after incubation. Absorbance of 490 nm was measured in each cell lysate, and melanin levels were calculated relative to those of control cells. (c) SK-MEL-28 cells (5ⅹ10^5^ cells/dish) were treated with 75, 150, and 300 µg/mL PME for 24 h. The protein levels of Tyrosinase, MiTF, p-p38, p38, COX2, and 𝛽-actin were measured by western blotting. Representative blots are shown. (d-e) SK-MEL-28 cells (5ⅹ10^5^ cells/dish) were pre-treated with 10 µM SB 203580 (SB) for 1 h, and then treated with 300 µg/mL of PME for 24 h. (d) Representative images of cell pellets of SK-MEL-28 cells after incubation. The melanin content (d) in cell lysates were analyzed by measuring the absorbance at 490 nm, and melanin levels were calculated relative to those of control cells. (e) The protein levels of COX2, tyrosinase and MiTF were measured by western blotting. Representative blots are shown. Data are presented as the mean ± SEM. ∗p<0.05, and ∗∗p<0.01 vs. control or PME.
